# Autologous cell therapy – A new concept to eradicate inhibitors in haemophilia

**DOI:** 10.1111/hae.14663

**Published:** 2022-09-19

**Authors:** Hanjing Xie, Rolf Ljung, Jan Astermark, Terese Hylander

**Affiliations:** ^1^ Idogen AB Medicon Village Scheelevägen 2 Lund Sweden; ^2^ Department of Oncology‐Pathology Karolinska Institute Stockholm Sweden; ^3^ Department of Clinical Sciences Lund University Lund Sweden; ^4^ Department of Paediatrics Lund University Lund Sweden; ^5^ Department of Translational Medicine Lund University Lund Sweden; ^6^ Department for Hematology, Oncology and Radiation Physics Skane University Hospital Malmö Sweden

Approximately 30% of patients with severe haemophilia A (HA) and 10% with severe haemophilia B (HB) develop neutralizing antibodies (inhibitors) towards Factor VIII (FVIII) and Factor IX (FIX), respectively, which are the main problems today in the treatment of haemophilia. Immune tolerance induction (ITI) based on various models using repeated administration of FVIII/FIX is the only proven method to eradicate inhibitors. However, ITI treatment fails in one‐third of the patients with HA and in more than half of those with HB. The recently registered, or currently under investigation in clinical trials, non‐factor products offer excellent protection against bleeding but will not reduce the frequency of inhibitors or induce immune tolerance. Tolerance to FVIII or FIX should remain the ultimate goal in haemophilia care to optimize the outcome and to enable the use of all current and future treatment options in all patients, but new strategies are needed urgently.

Idogen AB, a Swedish biotechnology company, has developed ItolDC‐028 which constitutes an autologous tolerogenic dendritic cell (DC) therapy for the treatment of patients with HA and inhibitors, in particular those who have failed to respond to conventional ITI (Figure 1). DCs are professional antigen‐presenting cells with a pivotal role in regulating immune responses. An important subset of DCs, the tolerogenic DCs, are key for establishing and maintaining tolerance and mediate suppression against self‐ and non‐pathogenic antigens. The development of DC‐based cell therapies is clinically attractive: not least through their potential of possessing low toxicity as they make use of the body's natural mechanisms for inducing and controlling immune tolerance to self‐antigens. Located at the apex of the immune responses, DCs simultaneously regulate the function of several effector cells that contribute to autoimmune‐related responses. ItolDC‐028 is manufactured using the patient's own (autologous) cells. As a first step, monocytes are collected from the patient using leukapheresis and the cells are thereafter shipped to the clinical manufacturing site. The monocytes are differentiated towards a stable dendritic cell phenotype with tolerogenic functions using a specific mix of tolerance‐inducing substances that act synergistically. At the end of the culture period the differentiated tolerogenic DCs are loaded with the FVIII antigen (recombinant full‐length FVIII). Finally, the cells are cryopreserved and this cell product, consisting of cryopreserved FVIII‐loaded tolerogenic DCs, is designated ItolDC‐028. In the clinic, ItolDC‐028 is thawed at the bedside and administered directly to the patient through intravenous (IV) injection. After IV injection, ItolDC‐028 will reach lymphoid organs, such as the liver and spleen, where it is anticipated to induce antigen‐specific tolerance against FVIII by activation and induction of regulatory T cells (Tregs) and regulatory B cells (Bregs). The latter suppress FVIII‐specific effector T cells and prevents generation of FVIII‐specific neutralizing antibodies generated by plasma cells. By these means, ItolDC‐028 will instruct the immune system to tolerate FVIII and make it possible to treat the tolerized patients with FVIII when required.

**FIGURE 1:**
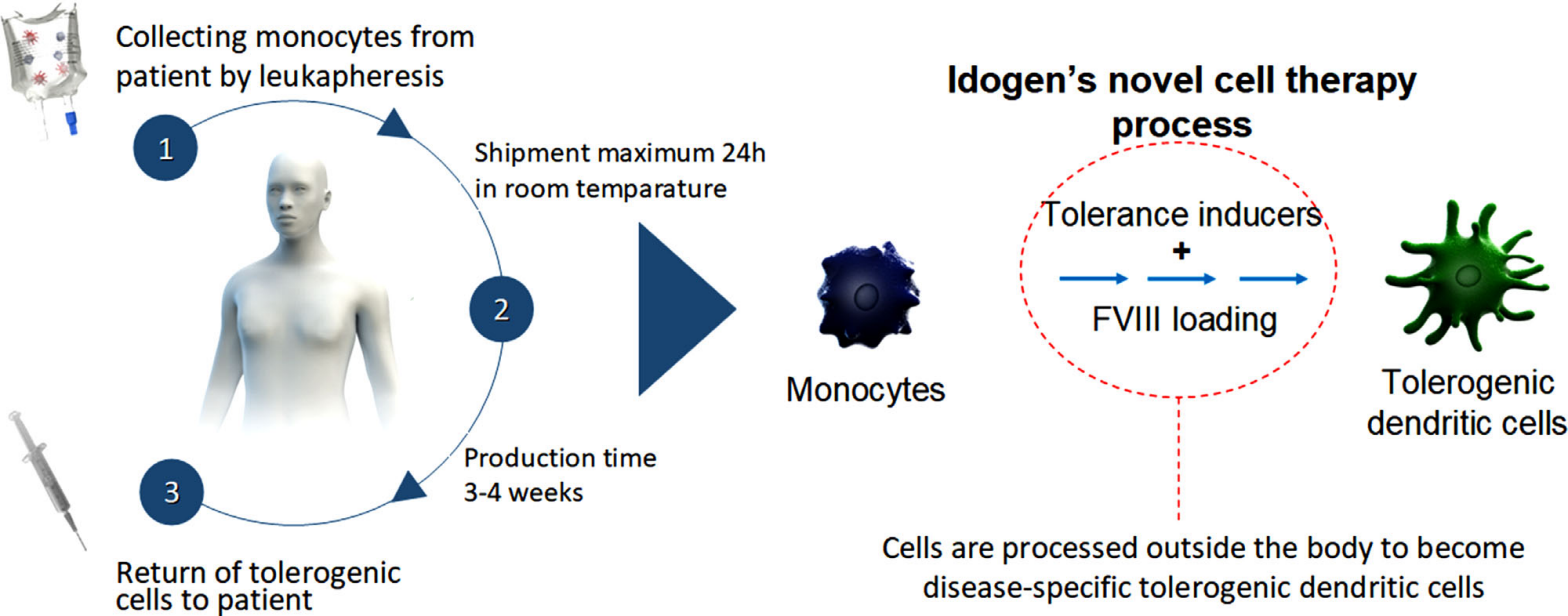
Overview of ItoDC‐028 cell therapy process.

Idogen's own in vitro and in vivo nonclinical data and other autologous tolerogenic DC therapies support the first‐in‐human clinical trial with ItolDC‐028 (manuscript in preparation, data available on request). As a result of the lack of an established surrogate animal model of ItolDC‐028, and since no appropriate animal‐based disease model therefore can be used, the nonclinical package is supported primarily by in vitro data. A comprehensive summary of these studies showed that ItolDC‐028 acquires a strong tolerogenic phenotype with low expression of activation markers. ItolDC‐028 has the capacity to attenuate the proliferation of allogeneic T cells and increase numbers of Tregs. The capacity of ItolDC‐028 to induce tolerance in vitro was equivalent, or better, compared with other tolerogenic DCs used previously in published clinical trials. In addition, ItolDC‐028 is stable and does not change phenotype or function after inflammatory challenge. To evaluate the safety/toxicity of IV administration of ItolDC‐028, a dose range toxicity study and a pivotal multiple dose toxicity study has been performed using immunocompromised mice. At the maximum dose administered to the mice, corresponding to 63 times the starting dose level in the clinical trial, there were no ItolDC‐028‐ related toxicological findings, no changes in safety parameters (including coagulation parameters) and no evidence of systemic thromboembolism. In addition, no local reactions were observed and no ItolDC‐028‐related macroscopic or microscopic changes in the organs (manuscript in preparation, data available on request).

Accumulating evidence continuously unravels the intricate processes involved in immune activation and tolerance induction. Such insights have inspired the development of adoptive cell therapy concepts based on a diversity of cells including both regulatory myeloid (regulatory macrophages) and lymphoid cells (regulatory T cells) that can initiate and drive tolerance and inhibit unwanted immune reactions long‐term.^1^ In addition, several publications report encouraging results in establishing tolerance using tolerogenic DCs and in patients with a number of autoimmune diseases including rheumatoid arthritis, diabetes type 1, Crohn's disease and multiple sclerosis.^2^, ^3^, ^4^, ^5^, ^6^, ^7^, ^8^ However, to date there are no approved tolerogenic cell therapies available for the treatment of HA patients with inhibitors.

Idogen's tolerogenic DCs have been developed as a novel cell platform technology with the aim of treating conditions with unwanted immune cell activation. The same tolerogenic cellular platform developed to restore tolerance towards FVIII in HA patients with inhibitors can potentially, with only minor adjustments to the manufacturing process, be transferred to target indications in which disease progression is caused by a single or multiple antigens.

No clinical data on the use of ItolDC‐028 are available to date. The upcoming first‐in‐human ItolDC‐028‐01 is an open‐label, multi‐centre, phase 1/2a trial to evaluate the safety and preliminary efficacy of ItolDC‐028 in patients with HA and inhibitors. The eligible patients are male, aged between 18‒60 years, diagnosed with congenital severe HA and with detectable neutralizing anti‐FVIII antibodies of a reported historic peak titre of BU >5 and BU >1 at inclusion. Additional fulfilment criteria include at least one confirmed ITI failure or classified as unsuitable for ITI administration. Patients with ongoing treatment with emicizumab will be allowed but not those treated with FEIBA (factor eight inhibitor bypassing activity). The primary endpoint of the trial is safety. The secondary endpoint is preliminary efficacy of ItolDC‐028 by evaluating the success of ITI as well as cell therapy‐induced immune responses.

The trial will employ a classic 3+3 design, with single escalating IV doses administered to a minimum of nine individuals at three levels: 36 × 10^6^, 72 × 10^6^ and 144 × 10^6^ cells. Additional subjects may be enrolled upon reported signs of dose‐limiting toxicities. Leukapheresis will be performed 4 weeks prior to the administration of ItolDC‐028. Patients will be challenged with FVIII (50 IU/Kg) 20 weeks after dosing with ItolDC‐028. The parameters for safety and preliminary efficacy evaluations will be assessed during the study conduct period of 26 weeks, but all patients will be followed up to 10 years in a follow‐up study. The main purpose of the current as well as and subsequent trials with ItolDC‐028 is to develop a therapy that makes the patient tolerant to FVIII and thus eliminates the problem of the immune system reacting with the production of neutralizing antibodies. This trial is planned to be carried out at 5–8 haematology sites in Europe. If the trial is successful in patients with inhibitors, a future development may be to adapt the concept with the aim of preventing the risk of developing neutralizing antibodies in the individual's first treatments with FVIII.

## AUTHOR CONTRIBUTION

All four authors participated in the planning of the manuscript and wrote selected parts which were then discussed and merged into the final version.

## DISCLOSURES

Hanjing Xie works on consultancy basis as Chief Medical Officer for Idogen AB; Rolf Ljung works on consultancy basis as a medical advisor to Idogen AB and Jan Astermark works on consultancy basis as a medical advisor to Idogen AB and will be the Principal Investigator in the clinical trial.

Rolf Ljung has during the last 3 years received compensation for consultancy work (DMC, Advisory Board) or remuneration for lectures from SOBI, Novo Nordisk, Takeda, Sanofi, Roche and Idogen AB.

Jan Astermark has received research grants from Sobi, CSL Behring, Takeda/Shire and Bayer, and speaker's fees and consultancy for Octapharma, Novo Nordisk, Pfizer, Bayer, Sobi, CSL Behring, Takeda/Shire, Idogen, BioMarin, Uniqure and Spark Therapeutics.

## Data Availability

Data sharing not applicable – no new data generated, or the article describes entirely theoretical research.
